# IL-17 producing T cells correlate with polysensitization but not with bronchial hyperresponsiveness in patients with allergic rhinitis

**DOI:** 10.1186/2045-7022-4-3

**Published:** 2014-01-15

**Authors:** Vanya M Tsvetkova-Vicheva, Svetla P Gecheva, Regina Komsa-Penkova, Angelika S Velkova, Tcvetan H Lukanov

**Affiliations:** 1 Department of Clinical Laboratory, Clinical Immunology and Allergology, Medical University of Pleven, Pleven, Bulgaria; 2 Department of Social and preventive medicine, Medical statistics, Pedagogy and psychology, Medical University of Pleven, Pleven, Bulgaria; 3 Department of Chemistry and Biochemistry, Medical University of Pleven, Pleven, Bulgaria

**Keywords:** IL-17, Th17, IL-4, IL-13, Allergic rhinitis, Atopy, Bronchial hyperresponsiveness

## Abstract

**Background:**

Th2-type T cell response has a considerable role in atopic diseases. The involvement of Th17 and IL-17 in atopy process provided new understanding of allergic diseases. Bronchial hyperresponsiveness is quite common in allergic rhinitis. We aimed to explore the expression of IL-17 producing CD3^+^ CD4^+^ T cells in peripheral blood of rhinitic patients, with/without bronchial hyperresponsiveness and sensitized to common allergens, as this relationship has not been examined.

**Methods:**

Sixty one patients with allergic rhinitis and thirty controls were examined. IL-17 producing T cells were detected by flow cytometry, IL-17, IL-4 and IL-13 levels in peripheral blood were evaluated by ELISA. Bronchial hyperresponsiveness was investigated with methacholine challenge test. Atopy was evaluated by skin prick tests with common allergens.

**Results:**

IL-17 producing T cell percentage of AR group was significantly higher: 2.59 ± 1.32 than in controls 1.24 ± 0.22, (p = 0.001). Significant sex related difference in CD3^+^ CD4^+^ IL-17 T cells was observed: respectively in male patients versus female 3.15 ± 1.8% and 2.31 ± 0.9%, (p = 0.02). Rhinitics had greater bronchodilator responses compared to controls (p = 0.001), however the percentages of T cells in both groups appeared equal. Serum IL-17 levels in AR group were significantly higher (5.10 ± 4.40) pg/ml than in controls (3.46 ±1.28) pg/ml, (p = 0.04). IL-4 levels (0.88 ± 1.27) and IL-13 levels (3.14 ± 5.85) in patients were significantly higher than in control’s (0.54 ± 0.10) pg/ml, (p = 0.001) and (1.19 ± 0.64) pg/ml; (p = 0.001) respectively.

The percentages of T cells in patients sensitized to 5 allergens (group I) were significantly lower (1.91 ± 0.62) than those sensitized to more than 5 allergens (group II) (2.91 ± 1.5) (p = 0.004).

**Conclusions:**

The observed higher levels of IL-17 producing T cells in polysensitized males suggest a role of IL-17 in pathogenesis of AR. The higher airway responsiveness in AR may not be Th17 dependent. The higher serum values of IL-17, IL-4 and IL-13 demonstrate the presence of cytokine balance in atopic diseases.

## Background

A trend of increasing prevalence of allergic rhinitis (AR) worldwide keeps the interest in investigation of pathogenesis of the disease. Th2 cells are suspected as the key culprit in the pathophysiology of atopic disorders [1]. Interleukin 17A (IL-17A), commonly known as IL-17, is produced by T helper 17 (Th17) subset of CD4^+^ T cells.

Recent progress in Th17 cells knowledge suggests a pathological role of IL-17 in the allergic responses [2]. However, the involvement of Th17 cells in AR has not been clearly examined [3]. Th2 cells produce IL-4 and IL-13 and mediate allergic responses and these cytokines have been extensively studied as key players in the atopic airway diseases. The roles of IL-4 in IgE production and IL-13 in bronchial hyperresponsiveness (BHR) and tissue remodeling are evident [1]. Th17 cells and IL-17 as a major family cytokine are usually associated with autoimmune reaction or neutrophil inflammation. Nevertheless it has been demonstrated that allergic sensitization through the airway promotes strong Th17 response and acute BHR in mouse model of asthma [4]. Asymptomatic airway hyperreactivity is common in people with AR, but the immunological mechanisms have to be defined. There are no data presenting the intimate role of Th17 cells in these events. Based on this, we hypothesized that IL-17 may mediate airway hyperresponsiveness in AR patients.

The present study examined the immunologic characteristics of sensitized patients with allergic rhinitis with/without bronchial hyperesponsiveness in comparison with healthy controls.

## Methods

### Subjects

Sixty one subjects (41 females and 20 males, mean age 36.25 ± 5 years) with persistent moderate-to-severe allergic rhinitis and thirty healthy controls (18 females and 12 males, mean age 35.73 ± 6 years) were included in the study. The subjects were selected by detailed clinical history of nasal obstruction, and/or rhinorrhea, sneezing, itching and sensitization to one perennial allergen at least. Selected patients experienced these symptoms since at least two years. Exclusion criteria were: bronchial asthma, chronic rhinosinuitis, nasal polyposis, excessive septal deviation and current smoking.

Blood samples for assessment of cytokines, IL-17 producing T cells and peripheral eosinophil counts were taken from patients and controls. The study was approved by the ethic committee of MU-Pleven, Bulgaria. Informed consent was obtained from all the subjects.

### Atopy assesment

The skin prick tests (SPT) were performed to patients and controls according to the method of Pepys [5].

The patients were not under antihistamine treatment since at least 10 days before skin prick tests. The following allergens (Stallergens) were used: House dust mites: (***dermatophagoides pteronyssinus****,* ***dermatophagoides farinae****)*, **Animal feather** (*feathers mixture*: duck, goose, hen), **cat**, **dog**, Grass mix (***3 grasses***: *dactylis glomerata, lolium perenne, phleum pratense*), Grass mix (***5 grasses***: *dactylis glomerata, lolium perenne, phleum pratense, anthoxanthum odoratum, poa pratensis* ), Grass mix (***4 cereals***: *avena sativa, triticum vulgare, zea mays, hardeum vulgare*), Grass mix **(*12 grasses****: holcus lanatus, festuca elatior, cynodon dactylon, bromus hordeaceus, avena fatua, arrhenatherum elatius, agrositis vulgaris, dactylis glomerata, lolium perenne, phleum pratense, anthoxanthum odoratum, poa pratensis*), Trees: **Beech** (*Fagaceae: castanea vulgaris, quercus robur, fagus sylvatica*)*,***Birch** (Betulaceae: *alnus glutinosa, betula alba, carpinus betulus, corylus avellona*), **Willow** (Salicaceae: *populus alba, salix caprea*), Weeds (***Ragweed, artemisia vulgaris***), Molds (***alternaria alternata, aspergillus mix, fusarium solani, penicillium mix***) and **Cockroach**. Histamine solution (10 mg/mL) and saline served as positive and negative control respectively. The size of the wheal was measured after 15 min. Diameter of ≥3 mm was considered as a positive reaction.

### Direct bronchial challenge testing with methacholine

Methacholine provocation test was performed according to the 2-min tidal breathing method and inhalation of an aerosol from a nebulizer at an output of 0.13 mL/min was used. Saline was used as control. The concentrations of methacholine from 0.03 to 16 mg/ml [6] were used. Forced expiratory volume for one sec (FEV1) was measured at 30 and 90 sec after completed two minutes inhalation preceded by baseline spirometry. An acceptable-quality FEV1 was obtained at each time point. The test was discontinued when a drop of 20% in FEV1 occurred. The protocol included a post-bronchodilator test. The patients and controls were consistent with the following criteria: nonsmokers and FEV1 ≥80% of predicted values before testing.

A modified version of English Wright nebulizer and Spirometer “Spirovit sp-10” (Schiller, Switzerland) were used.

### Expression of IL-17A in peripheral blood T lymphocytes

#### *Blood sample collection and PMBC culture*

Blood samples from studied subjects were collected in EDTA vacutainer (Becton Dickinson, USA) and used for peripheral blood mononuclear cells (PBMC) isolation.

PMBC were obtained by centrifugation on Ficoll-Paque Plus (GE Healthcare, Sweden) and resuspended in 10% FBS-RPMI 1640 (Invitrogen). Cells were cultured in presence of 50 ng/ml Phorbol Myristate Acetate (PMA) (Sigma) for 5 hours at 37°C. After the first hour of incubation a protein transport inhibitior (GolgiPlug, Becton Dickinson) was added followed by consequent incubation and washing procedures.

### Intracellular staining of IL-17A cytokine

Cells were stained for cell surface markers with Anti-Human CD3 PerCP (Becton Dickinson) and MAB FITC-anti-human CD4 (Becton Dickinson) followed by fixation and permeabilization with Cytofix/Cytoperm. MAB PE anti-human IL-17A (Becton Dickinson Pharmingen) for the intracellular cytokine staining was used. After washing with PBS and 1% formaldehyde fixation, blood samples were stored at 40C prior to flow cytometry. Isotype control was included in each assay. Flow cytometer FACSort model (Becton Dickinson) was used. Results were expressed in percentages.

### In vitro determination of IL-4, IL-13 and IL-17

#### *Specimen collection*

Five ml of peripheral blood were collected using pyrogen/endotoxin free collecting tubes from all subjects (patients and controls). Serum was removed rapidly and carefully from the red cells after clotting followed by centrifuge at approximately 1000 × g for 10 min.

IL-4, IL-13 and IL-17 levels in serum were analyzed using commercially available pre-coated enzyme-linked –immunosorbent –assay (ELISA) kits (Diaclone SAS, France). All reagents were brought to room temperature before use. Preparation of Wash Buffers, Standard Diluent Buffers and Controls followed the instructions in the manufacturer’s manual. After the final preparation of all reagents, the average absorbance values for each set of triplicate standards, controls and samples were calculated within 20% of the mean. A linear standard curve was generated. The amounts of IL-4, IL-13 and IL-17 in each sample were determined by extrapolating OD values using a standard curve. The lower limit of detection was <0.54 pg/ml for IL-4, <1.17 pg/ml for IL-13 and <3.2 pg/ml for IL-17.

#### *Statistical analysis*

Variables with normal distribution were expressed as mean and standard deviation.

For comparison of two independent groups the non-parametric Mann–Whitney U test was used.

Statistical analysis was performed using SPSS (version 19.1). The results were expressed as mean ± standard deviation (SD). P <0.05 was considered as significant.

## Results

### Demographic characteristics of study population

A total of 91 subjects (61 patients and 30 controls) age matched were included in the study. Demographic data of AR group and controls are presented on Table 1, as well as eosinophil (Eo) count and BHR.

**Table 1:** Baseline characteristics of study subjects

	**Controls**		**AR patients**		**Total**	**p**
N	30		61		91	
Gender (female/male)	18/12		41/20		59/32	
Age (year)	35.73 ± 6		36.25 ± 5		36.08 ± 6	
Eo count %	2.32 ± 1.84		4.48 ± 2.13			0.001
BHR n	7 (Mch+)	23 (Mch-)	41 (Mch+)	20 (Mch-)		0.001
(%)	(23.3)	(76.7)	(67.2)	(32.8)		

### IL-17 producing T cells in peripheral blood were higher in AR patients

**Figure 1:**
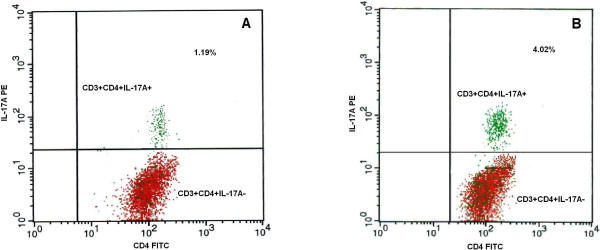
**PMBCs isolated from controls and allergic rhinitis patients were stimulated with PMA and Brefeldin A for 5 hours followed by adding MAB FITS anti-human CD4 and anti-human CD3 PerCP.** Production of IL-17 by PBMCs was determined at the single-cell level by intracellular cytokine staining and flow cytometric analysis. The upper right quadrants represent the percentage of IL-17 T cells with expression of CD3 and CD4 cells in the total lymphocyte populations gated. The dot plots show CD4 T cells producing IL-17 in human peripheral blood lymphocytes from healthy controls **(A)** and rhinitics **(B)**.

Average percentages of IL-17 producing T cells determined with flow cytometer were between 0.57 to 1.84% in healthy subjects and 1.34 to 6.84% in the patients (Figure 1).

**Figure 2:**
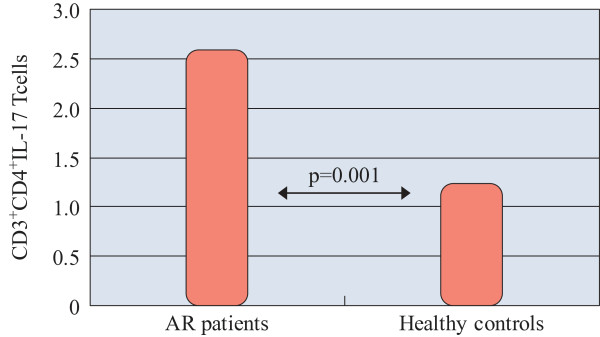
**IL-17 producing CD4**^**+ **^**T cells in AR patients and control subjects measured by flow cytometry (%).**

The percentage of IL-17 producing T cells in PMBC from whole blood of allergic rhinitis patients was significantly increased (2.59 ± 1.32%) as compared to controls (1.24 ± 0.22%) (p = 0.001) (Figure 2).

### IL-17 producing T cells were higher in males in both groups of rhinitics and controls

**Figure 3:**
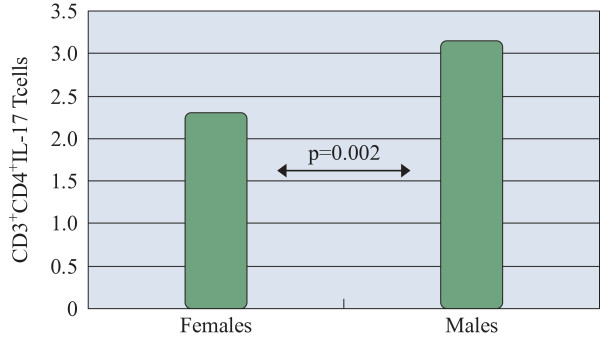
**IL-17 producing CD4**^**+ **^**T cells in male and female AR patients measured by flow cytometry (%).**

The gender related experimental outcome was assessed. In the study, 64.8% of the subjects were women. The percentages of CD3^+^ CD4^+^ IL-17 T cells were significantly higher in male patients: 3.15 ± 1.81 versus 2.31 ± 0.91 in females (p = 0.02) (Figure 3).

### IL-17 expression was dependent on sensitization rate

Table 2 presents the relationship between average percentages of Th17 expressing IL-17 and different perennial (indoor) allergens in patients and controls. Table 3 shows identical correlation with outdoor allergens (Table 3).

**Table 2:** Mean percentages of IL-17 producing T cells in patients and controls, measured by flow cytometry, corresponding with inhalant perennial allergens

**Allergen**	**Mean values of IL-17 producing T cells in patients (%)**	**Mean values of IL-17 producing T cells in controls (%)**
D. pteronyssinus*	2.59 ± 1.45	1.25 ± 0.00
D. farinae*	2.57 ± 1.26	1.24 ± 0.01
Animal feather	2.63 ± 1.33	1.23 ± 0.00
Cat	3.46 ± 1.7	1.31 ± 0.08
Dog	3.91 ± 1.88	1.25 ± 0.01
*Alternaria alternata*	3.04 ± 1.33	1.22 ± 0.06
*Aspergillus mix*	2.65 ± 1.10	0.00
*Fusarium solani*	2.22 ± 0.55	1.26 ± 0.00
*Penicillium mix*	3.17 ± 0.92	0.00
Cockroach	2.51 ± 1.08	1.3 ± 0.00

*p < 0.05.

There was no significant difference in percentage of IL-17 producing T cells referred to each parameter in allergen’s spectrum except *Dpt* and *D. Farinae*.

**Table 3:** Mean percentages of IL-17 producing T cells in patients and controls, measured by flow cytometry, corresponding with inhalant pollen allergens

**Allergen**	**Mean values of IL-17 producing T cells in patients (%)**	**Mean values of IL-17 producing T cells in controls (%)**
*3 grasses*	3.42 ± 1.61	1.22 ± 0.00
*5 grasses*	3.31 ± 1.62	1.22 ± 0.00
*4 cereals*	3.57 ± 1.63	1.22 ± 0.00
*12 grasses*	2.77 ± 1.05	1.22 ± 0.00
Birch	2.50 ± 0.82	1.22 ± 0.00
Beech	2.99 ± 1.33	0.00
Willow	3.87 ± 1.66	0.00
Ragweed	2.81 ± 0.65	0.00
*Artemisia vulgaris*	3.81 ± 2.20	0.00

**Figure 4:**
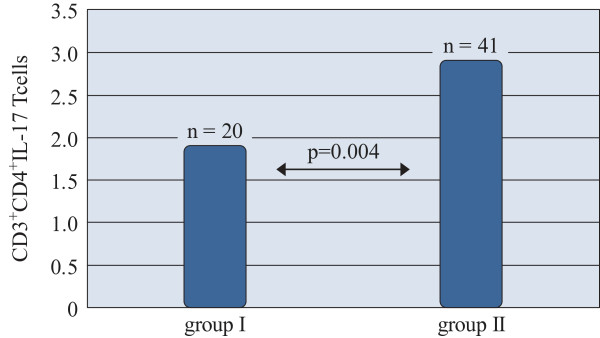
**IL-17 producing CD4**^**+ **^**T cells in patients sensitized to 5 allergens (group I) and rhinitics sensitized to more than 5 allergens (group II) measured by flow cytometry (%).**

In terms of skin-prick test results patients were assigned as: group I - sensitized to five allergens and group II - sensitized to more than 5 allergens. The average of IL-17 producing T cells percentages in two groups was significantly different: 1.91 ± 0.62% for group one and 2.91 ± 1.5% for group two (p = 0.004) (Figure 4).

### Increased levels of IL-4, IL-13 and IL-17 in AR patients

To elucidate the role of IL-17 in allergic inflammation in parallel with the classical participants (IL-4 and IL-13), all of them (IL-4, IL-13 and IL-17) were analyzed by ELISA method in the patients and controls.

**Figure 5:**
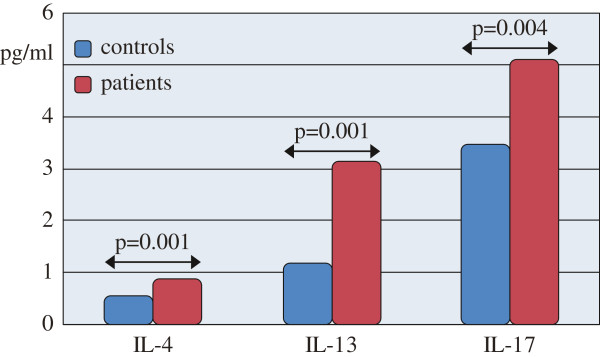
IL-4, IL-13 and IL-17 levels in allergic rhinitis patients and healthy subjects measured by ELISA.

The mean values in healthy subjects were within the normal ranges closely related to the available in the manufacturer’s manual. The mean levels in healthy controls were: 0.54 pg/ml (IL-4), 1.17 pg/ml (IL-13), and 3.2 pg/ml for (IL-17) as measured in our laboratory. The obtained values above these characters were accepted as pathological (Figure 5). The average values for IL-4, IL-13 and IL-17 levels were higher in patients compared to controls.

IL-4 levels were significantly higher (0.88 ± 1.27) pg/ml in patients versus (0.54 ± 0.10) pg/ml in controls, (p = 0.001).

The mean values were significantly higher for IL-13 in rhinitics (3.14 ± 5.85) pg/ml and (1.19 ± 0.64) pg/ml in healthy subjects, (p = 0.001).

The mean values of IL-17 were significantly higher in patients (5.10 ± 4.40) pg/ml as compared to asymptomatic subjects (3.46 ±1.28) pg/ml, (p = 0.04).

There was a significant difference between AR group and healthy subjects for all investigated interleukins.

Table 4 presents the association between each aeroallergen causing symptoms throughout the year and serum levels of IL-4, IL-13 and IL-17. The perennial allergens of house dust mites correlate significantly with the three interleukins. Animal feather allergen is strongly associated with IL-4 and IL-13.

**Table 4:** Mean values of IL-4, IL-13 and IL-17 in patients and controls, measured by ELISA, corresponding with inhalant perennial allergens

	**IL-4**		**IL-13**		**IL-17**	
**Allergen**	**Mean values of patients [pg/ml]**	**Mean values of controls [pg/ml]**	**Mean values of patients [pg/ml]**	**Mean values of controls [pg/ml]**	**Mean values of patients [pg/ml]**	**Mean values of controls [pg/ml]**
*D. pteronyssinus*	3.16 ± 2.73*	1.53 ± 2.09	3.21 ± 2.67*	1.38 ± 2.11	3.24 ± 2.77*	2.15 ± 2.46
*D. farinae*	2.31 ± 2.38*	1.30 ± 1.93	2.26 ± 2.32	1.38 ± 2.11	2.52 ± 2.43	1.61 ± 2.09
Animal feather	0.51 ± 1.36*	0.00	0.50 ± 1.35*	0.00	0.43 ± 1.36	0.28 ± 0.93
Cat	0.70 ± 1.99	0.20 ± 0.76	0.58 ± 1.91	0.44 ± 1.15	0.48 ± 1.66	0.63 ± 1.81
Dog	0.52 ± 1.70	0.33 ± 1.03	0.53 ± 1.71	0.31 ± 0.93	0.52 ± 1.77	0.43 ± 1.29
*Alternaria alternata*	0.72 ± 1.73	0.70 ± 1.64	0.77 ± 1.77	0.59 ± 1.55	0.45 ± 1.27	1.00 ± 2.02
*Aspergillus mix*	0.56 ± 1.47	0.20 ± 1.09	0.55 ± 1.46	0.21 ± 1.11	0.71 ± 1.64	0.22 ± 1.05
*Fusarium solani*	0.47 ± 1.35	0.27 ± 1.05	0.55 ± 1.46	0.10 ± 0.56	0.52 ± 1.47	0.33 ± 1.08
*Penicillium mix*	0.31 ± 1.07	0.00	0.31 ± 1.06	0.00	0.29 ± 1.07	0.15 ± 0.73
Cockroach	0.10 ± 0.54	0.23 ± 0.90	0.15 ± 0.65	0.14 ± 0.74	0.21 ± 0.78	0.09 ± 0.59

*p < 0.05.

The data of blood levels of the studied variables (IL-4, IL-13 and IL-17) and outdoor allergens are shown in Table 5. The average mean values of IL-4 showed significantly higher levels in patients sensitized to birch and beech pollens. IL-13 blood concentrations were significantly higher in beech sensitized rhinitics. There was no significant difference in IL-17 serum levels for outdoor allergens.

**Table 5:** Mean values of IL-4, IL-13 and IL-17 in patients and controls corresponding with inhalant pollen allergens (measured by ELISA)

	**IL-4**		**IL-13**		**IL-17**	
**Allergen**	**Mean values of patients [pg/ml]**	**Mean values of controls [pg/ml]**	**Mean values of patients [pg/ml]**	**Mean values of controls [pg/ml]**	**Mean values of patients [pg/ml]**	**Mean values of controls [pg/ml]**
*3 grasses*	1.95 ± 3.10	1.00 ± 2.41	1.85 ± 3.11	1.17 ± 2.41	2.31 ± 2.99	1.13 ± 2.81
*5 grasses*	1.67 ± 2.63	1.03 ± 2.53	1.60 ± 2.69	1.17 ± 2.41	2.02 ± 2.87	1.04 ± 2.33
*4 cereals*	1.79 ± 3.10	1.03 ± 2.53	1.73 ± 3.16	1.14 ± 2.37	1.98 ± 3.22	1.24 ± 2.71
*12 grasses*	0.36 ± 1.27	0.43 ± 1.36	0.48 ± 1.42	0.17 ± 0.93	0.52 ± 1.50	0.28 ± 1.11
Birch	1.69 ± 3.10*	0.50 ± 1.38	1.48 ± 3.02	0.90 ± 1.86	1.88 ± 3.32	0.78 ± 1.98
Beech	1.18 ± 2.28*	0.27 ± 1.05	1.19 ± 2.24*	0.21 ± 1.11	1.19 ± 2.21	0.65 ± 1.83
Willow	0.52 ± 1.70	0.00	0.44 ± 1.58	0.17 ± 0.93	0.31 ± 1.16	0.41 ± 1.65
Ragweed	0.16 ± 0.90	0.13 ± 0.73	0.16 ± 0.89	0.14 ± 0.74	0.21 ± 0.98	0.11 ± 0.74
*Artemisia vulgaris*	0.25 ± 1.15	0.00	0.24 ± 1.14	0.00	0.29 ± 1.31	0.07 ± 0.44

*p < 0.05.

Patients who were sensitized to birch and beech allergens showed significantly higher values of IL-4. IL-13 was significantly higher in patients with positive SPT to beech pollen.

### Allergic rhinitis patients were hyperresponsive to methacholine independently of IL-17 producing T cells

In comparison with healthy subjects, those with AR had a higher degree of bronchial responsiveness to methacholine challenge 67.2% versus 23.3% respectively (p = 0.001).

The average percentages of IL-17 producing T cells (CD3^+^CD4^+^) in methacholine positive and methacholine negative rhinitics appeared equal (p > 0.05).

The percentages of T cells in patients sensitized to 5 allergens (group I) were significantly lower (1.91 ± 0.62) than those sensitized to more than 5 allergens (group II) (2.91 ± 1.5) (p = 0.004).

## Discussion

This study shows that increased levels of IL-17 producing T cells may play role in the allergic inflammation in patients with AR. Our observations suggest that IL-17 production might be triggered by the increasing number of sensitizations. In the pathophysiology of allergic rhinitis, a sensitization process includes activation of allergen-specific T cells commonly presented by Th2 subtype. The specific T cells stimulate the production of allergen-specific IgE which is the essential issue in AR [7]. A recent study on IgE production in human B cells found that IL-17 could induce B cells switching to IgE which implies Th17 involvement in the atopic phenomenon [8]. The role of IL-17 producing cells as the subject of research in the field of allergic sensitization is essential not only for increasing our understanding of the AR mechanism. The process of polysensitization is associated with substantial impact on quality of life of rhinitis patients [9]. It has been reported that polysensitized subjects experienced more severe symptoms than monosensitized ones [9] and frequently presented associated asthma. These clinical implications make the recognition of IL-17 as being important in the pathogenesis of allergic rhinitis.

Furthermore, we examined IL-17 dependent BHR in AR patients for the first time. The results obtained show that subjects with BHR to methacholine exhibit similar percentages of IL-17 producing T cells as methacholine negative patients.

Yet, the subjects with asymptomatic BHR differed significantly from healthy controls.

It would be important to follow up the AR patients with BHR aiming to understand are they at greater risk of developing IL-17 dependent asthma symptoms within the succeeding few years.

The role of IL-13 in mediating lower airway inflammation and BHR following isolated allergic sensitization of the upper airways was suggested in a recent study of Wang et al. [10]. The authors highlighted the role of interleukins in the communication between the upper and lower airways. Notwithstanding the recent evidence suggesting IL-17 contribution to BHR [11], raised new and complex questions. Data for IL-17 mediated allergic rhinitis in humans are quite limited compared to murine experimental models. Moreover, recent studies have been demonstrated that cytokine setting inducing Th17 differentiation in humans and mice is different [12]. The same authors state the role of IL-17 producing cells in allergic reactions as “largely unclear”. In 2010 for the first time IL-17 have been found to play a causative role in airway remodeling in asthmatic mouse model [13]. But the idea on participation of IL-17 in upper airway tissue remodeling is not clear and is still controversial.

Furthermore, we demonstrate that rhinitic subjects display high levels of IL-4, IL-13 and IL-17 in peripheral blood. IL-4 and IL-13 are key players in the allergic response [1] as it was confirmed in our study also. Despite missing information on Th2 expression in this study, both IL-4 and IL-13 served as important indicators of Th2 expression. Recent studies demonstrated that both Th2 and Th17 are involved in the pathogenesis of allergic airway inflammation through releasing specific cytokines [14]. IL-13 is essential for the development of a late nasal response to allergen challenge [15] while IL-4 plays an important role in the early Th2 inflammatory response [1]. The Th17 subset discovery revised the Th1/Th2 paradigm and enhanced the understanding of heterogeneous nature of allergic disease [1].

The limitation of this study is relatively small number of control subjects experienced to methacholine and skin prick tests, which could be overcome in a future study.

## Conclusions

The higher levels of IL-17 producing CD3^+^CD4^+^T cells in peripheral blood of polysensitized allergic rhinitis patients were demonstrated.

The increased BHR in AR subjects and their airway response was suggested to enhance upon natural exposure to multiple allergens to which the subjects were sensitized.

The role in allergic inflammation of Th2 associated cytokines was confirmed.

Settings of high levels of IL-4, IL-13 and IL-17 in AR subjects on multiple allergen exposure support the idea of the heterogeneous nature of allergic disease and the role of Th1/Th2/Th17 balance in the pathogenesis of AR.

## Abbreviations

AR: Allergic rhinitis; BHR: bronchial hyperresponsiveness; SPT: skin prick test; Mch: methacholine; Gecheva P Svetla: Velkova Angelika S and Lukanov H Tcvetan contributed equally to this work.

## Competing interests

The authors declare that they have no competing interests.

## Authors’ contributions

VT-V conceived of the study and participated in the design of the study, recruited and phenotyped the subjects, analysed and interpreted the data and drafted the manuscript. SG and TL carried out flow cytometric assays. RK was involved in the data analysis and revising of the manuscript. AV performed the statistical analysis. All authors read and approved the final manuscript.
